# Beyond Dementia: Uncovering Superficial Siderosis in the Memory Clinic

**DOI:** 10.1192/bjo.2026.11870

**Published:** 2026-06-30

**Authors:** Dilshana Nafisa Bapakunhi, Bhavana Reddy

**Affiliations:** 1 Essex partnership University NHS Foundation trust, Colchester, United Kingdom; 2 Central and northwest London NHS Foundation trust, Colchester, United Kingdom

## Abstract

**Aims:**

Superficial siderosis of the central nervous system is a rare progressive disorder caused by chronic deposition of haemosiderin in the superficial layers of the brain and spinal cord, often following recurrent subarachnoid bleeding. The cerebellum and hindbrain structures are particularly vulnerable to iron-related toxicity. Although classically characterised by ataxia, dysarthria, and gait disturbance, cognitive impairment is increasingly recognised as part of its presentation. This overlap can obscure diagnosis, especially when patients first present to memory services with primarily cognitive complaints.

**Methods:**

A 75-year-old woman was referred to the memory clinic with a one-year history of worsening forgetfulness, difficulty recalling recent events, and episodes of disorientation. Her family reported rapid decline in her functional abilities, and she had become dependent on her son for daily organisation. She appeared anxious during cognitive testing and showed impaired short-term memory, word-finding difficulty, and repetitive speech, although reading and writing remained intact.

Initially, her presentation resembled a primary cognitive disorder. However, neurological examination revealed significant abnormalities, including difficulty following complex commands, tongue and limb apraxia, bilateral dysdiadochokinesia, and impaired tandem walking. Although there were no cranial nerve deficits or nystagmus, prominent cerebellar signs prompted neuroimaging. MRI demonstrated severe superficial siderosis symmetrically affecting the infratentorial region with lesser supratentorial involvement.

Her cognitive and neurological function deteriorated despite anti-dementia medication. A neurology opinion was sought, and she required multidisciplinary involvement, including speech and language therapy for dysphagia and fallsprevention support. As the condition progressed, she developed leftsided weakness, an upgoing plantar response, and reduced ankle reflexes. Chelation therapy with deferiprone was considered but not pursued due to advanced cognitive impairment, marked ataxia, and limited potential for benefit.

**Results:**

This case highlights an important diagnostic challenge for memoryassessment clinicians: superficial siderosis can initially present as a cognitive disorder, even though the underlying pathology is neurological. Without thorough neurological examination, such cases may be misdiagnosed as dementia, particularly when dysarthria and inconsistent cognitive testing obscure the picture. Dysarthria limits verbal responses, reduces the validity of cognitive scores, and can exaggerate perceived deficits. The patient’s apraxia, coordination abnormalities, and gait disturbance indicated a broader process inconsistent with typical neurodegenerative dementia. Early recognition is essential, not because curative therapy exists, but to guide appropriate imaging, avoid unnecessary treatments, initiate timely multidisciplinary care, and prepare families for progressive decline.

**Conclusion:**

Cognitiveled presentations of superficial siderosis are uncommon but clinically important. This case highlights the value of thorough neurological assessment in memory services, particularly when cognitive symptoms present with atypical neurological features.

